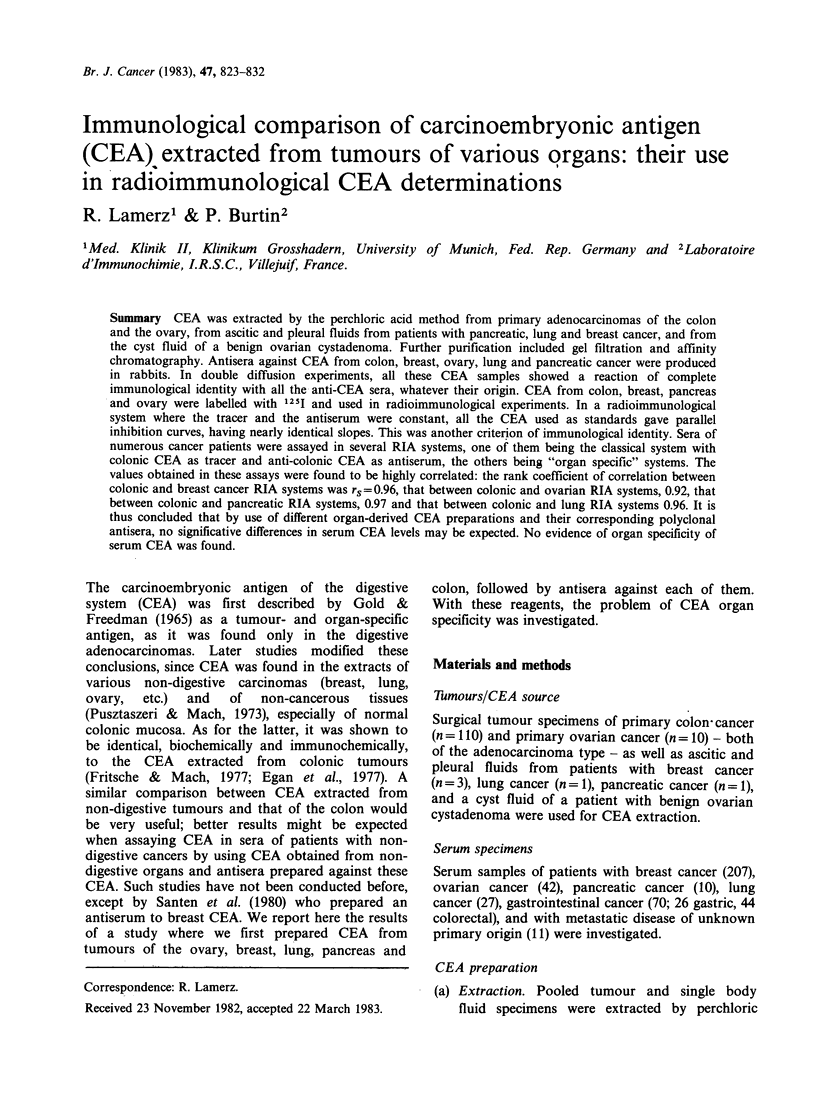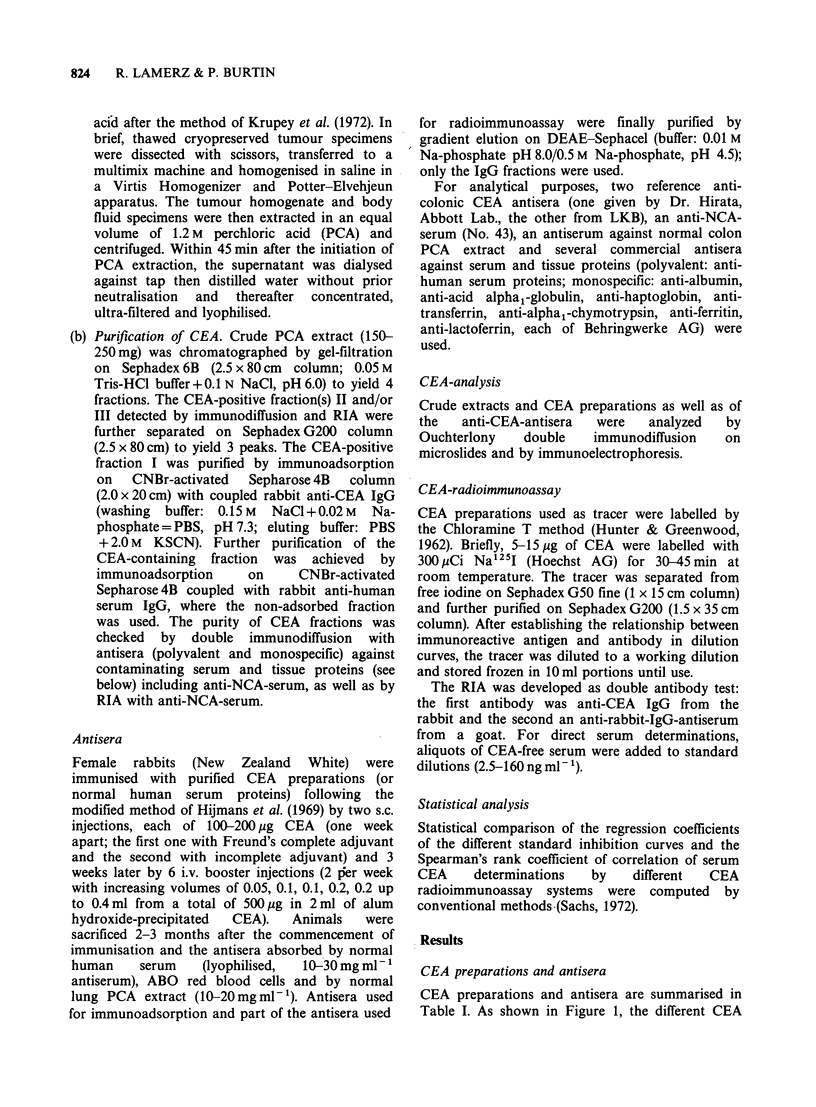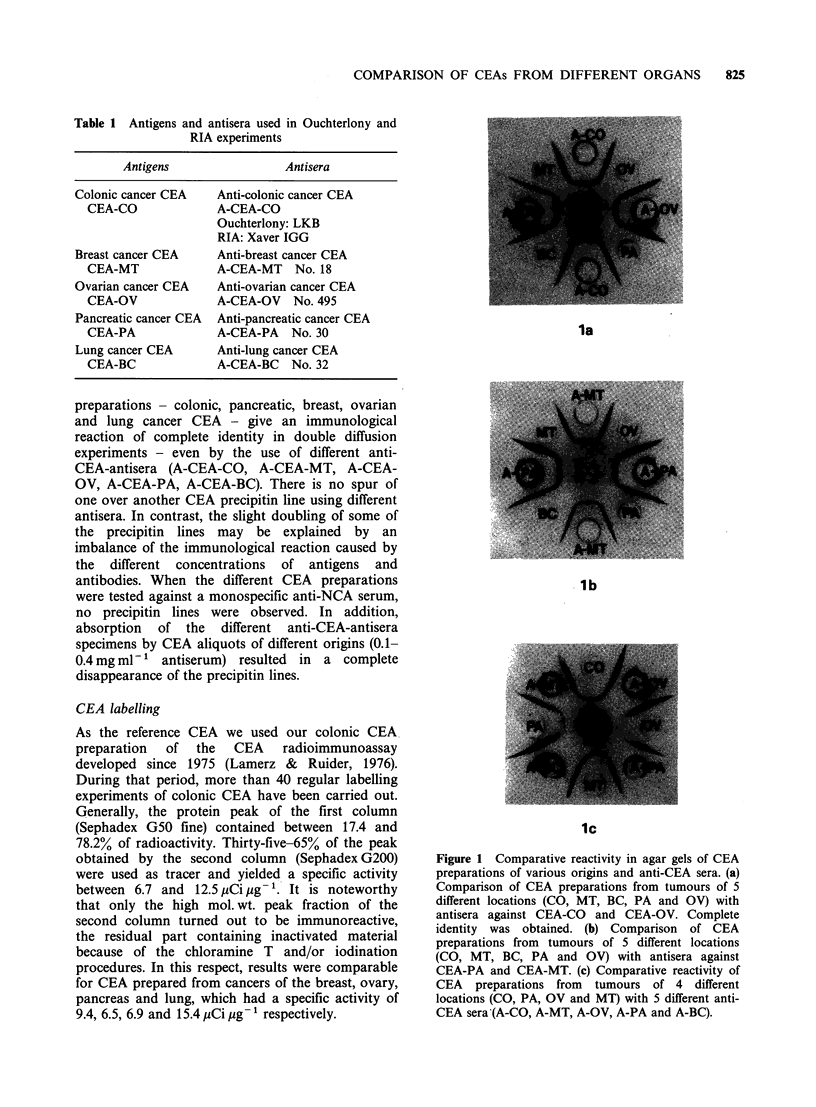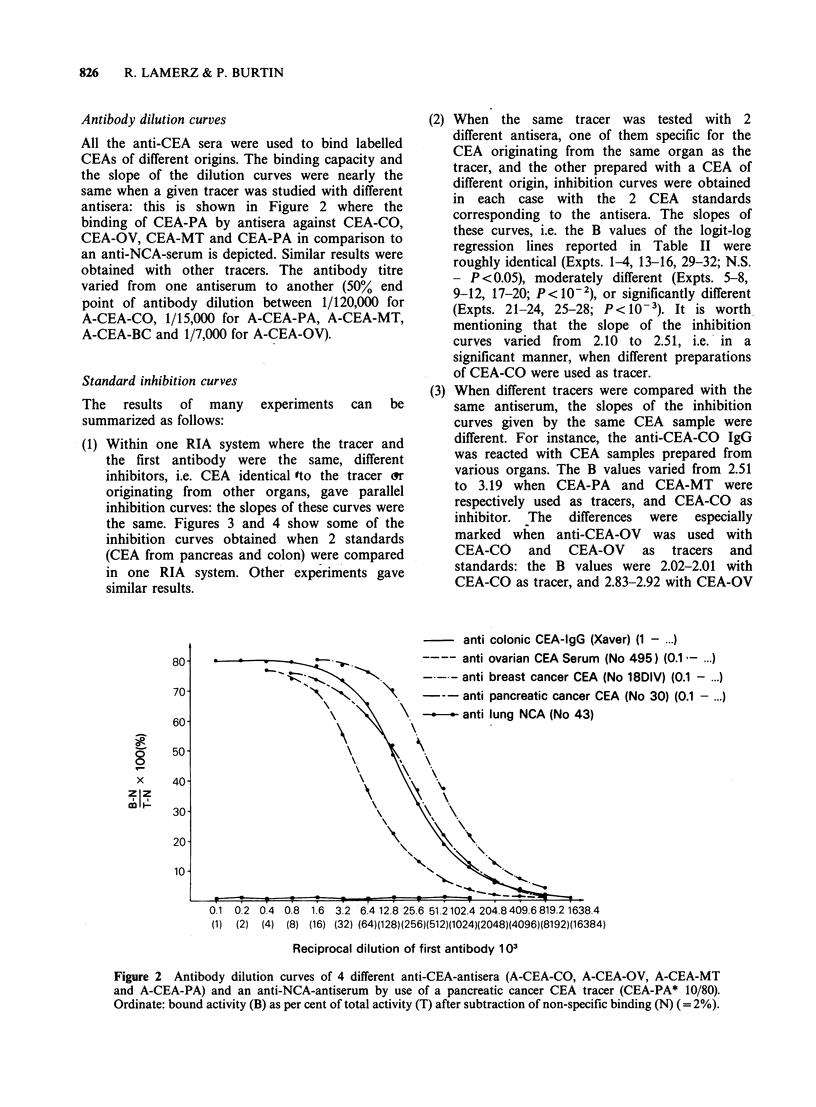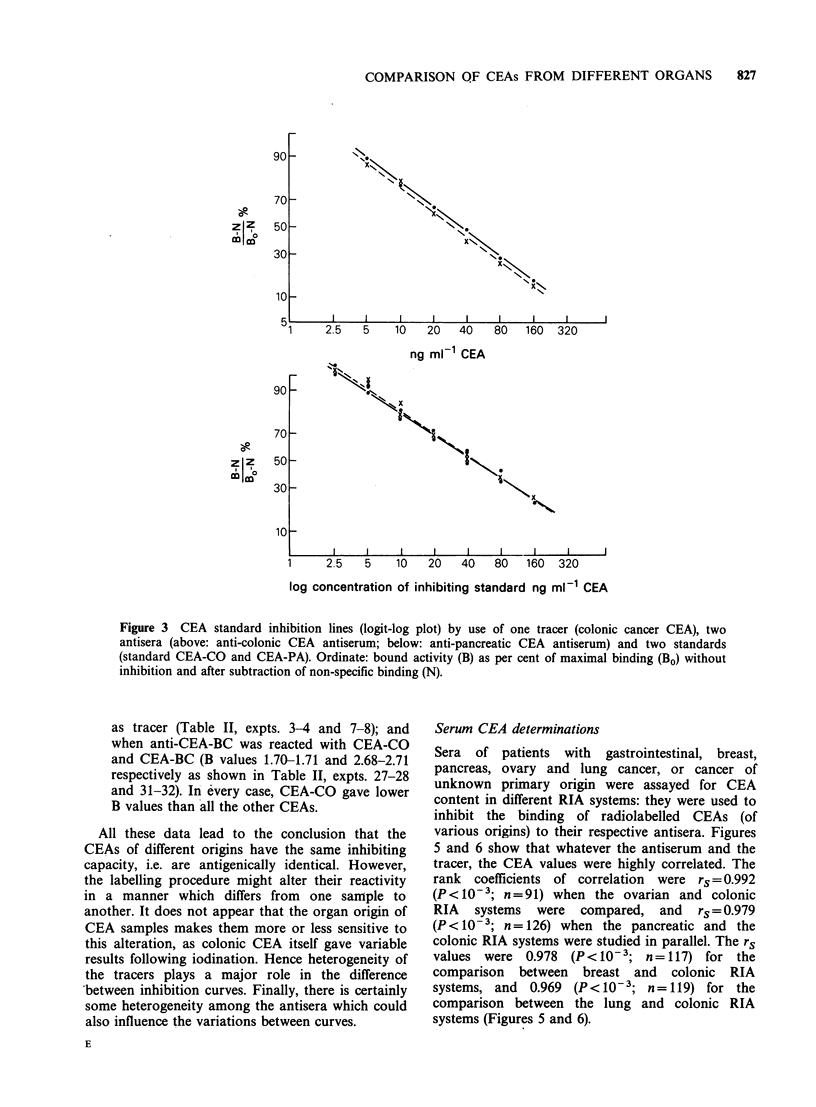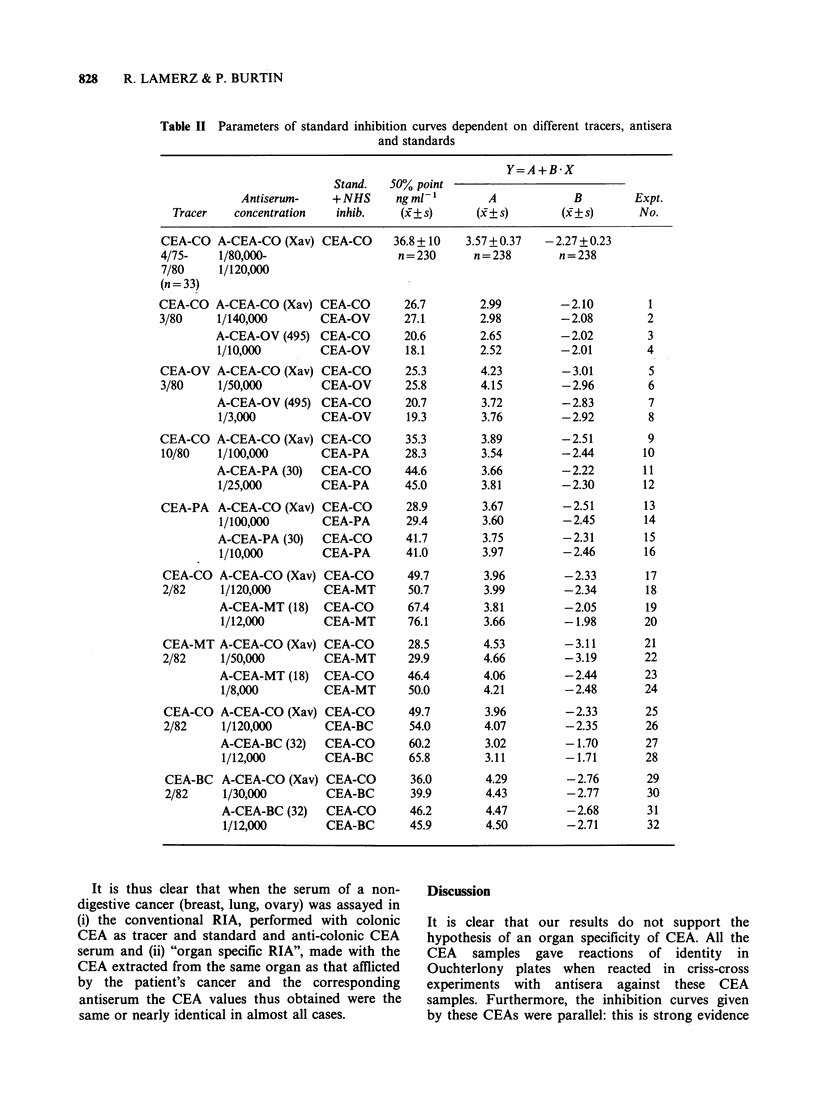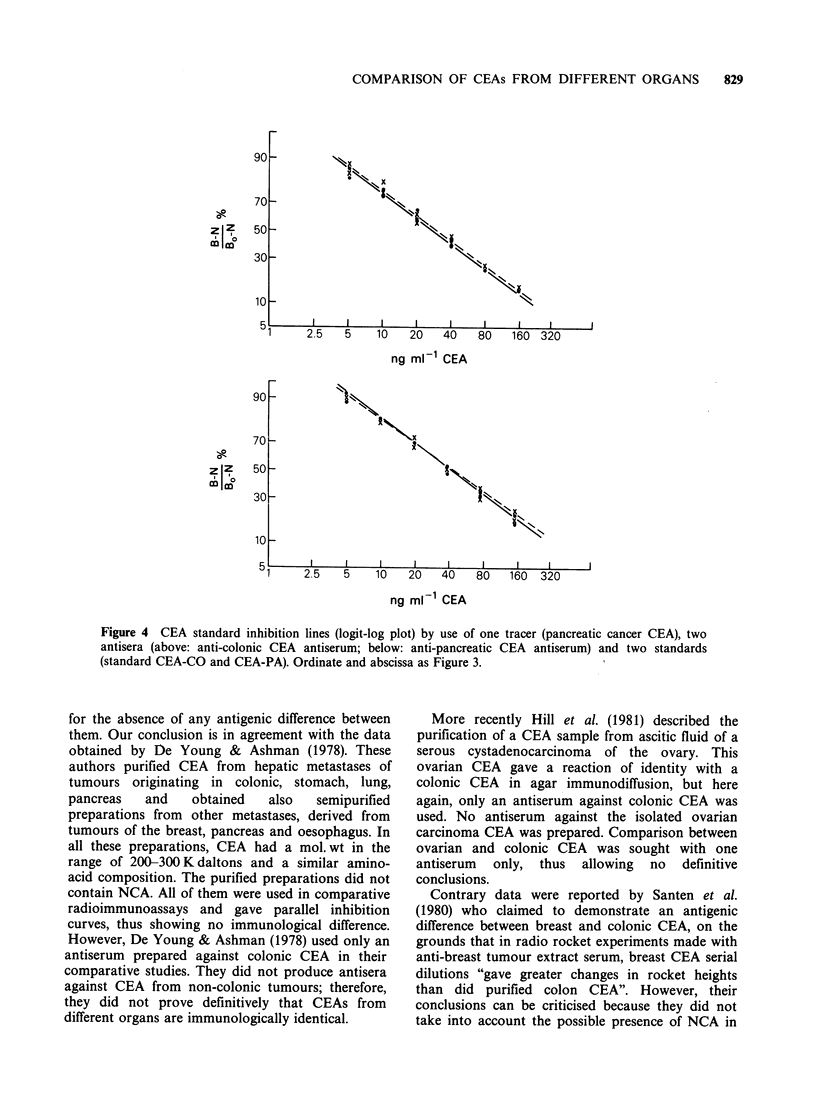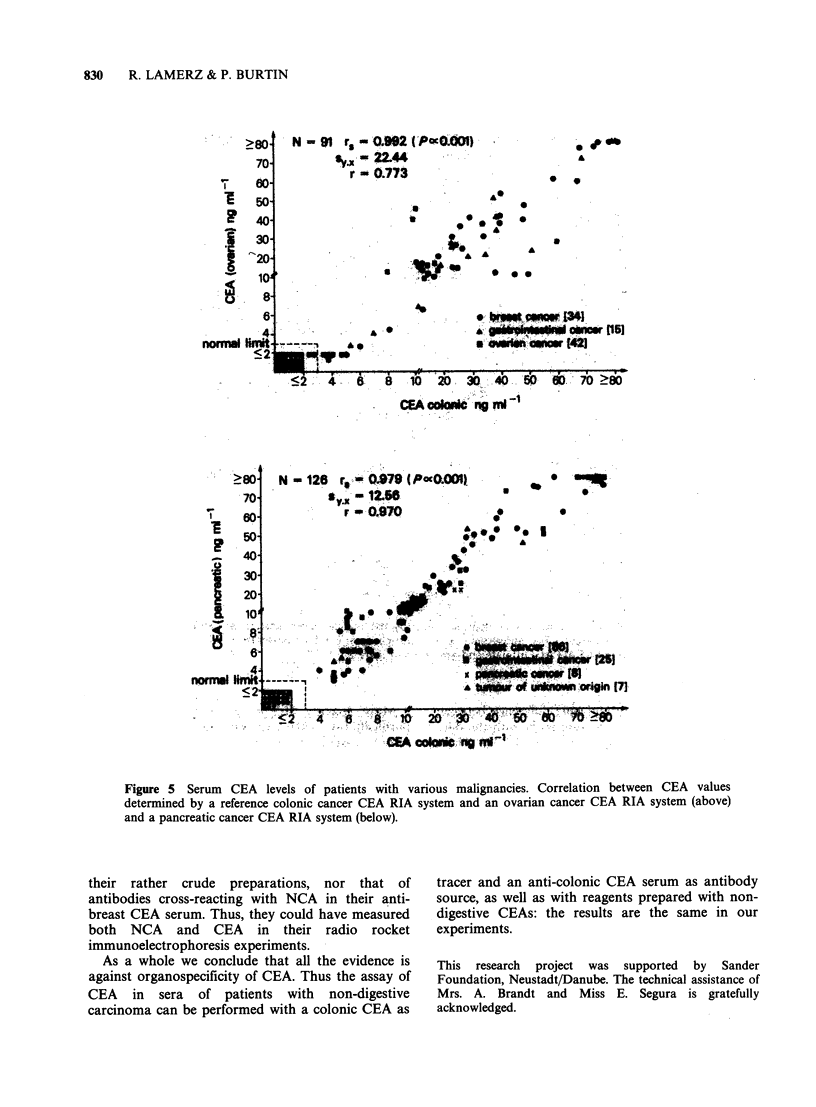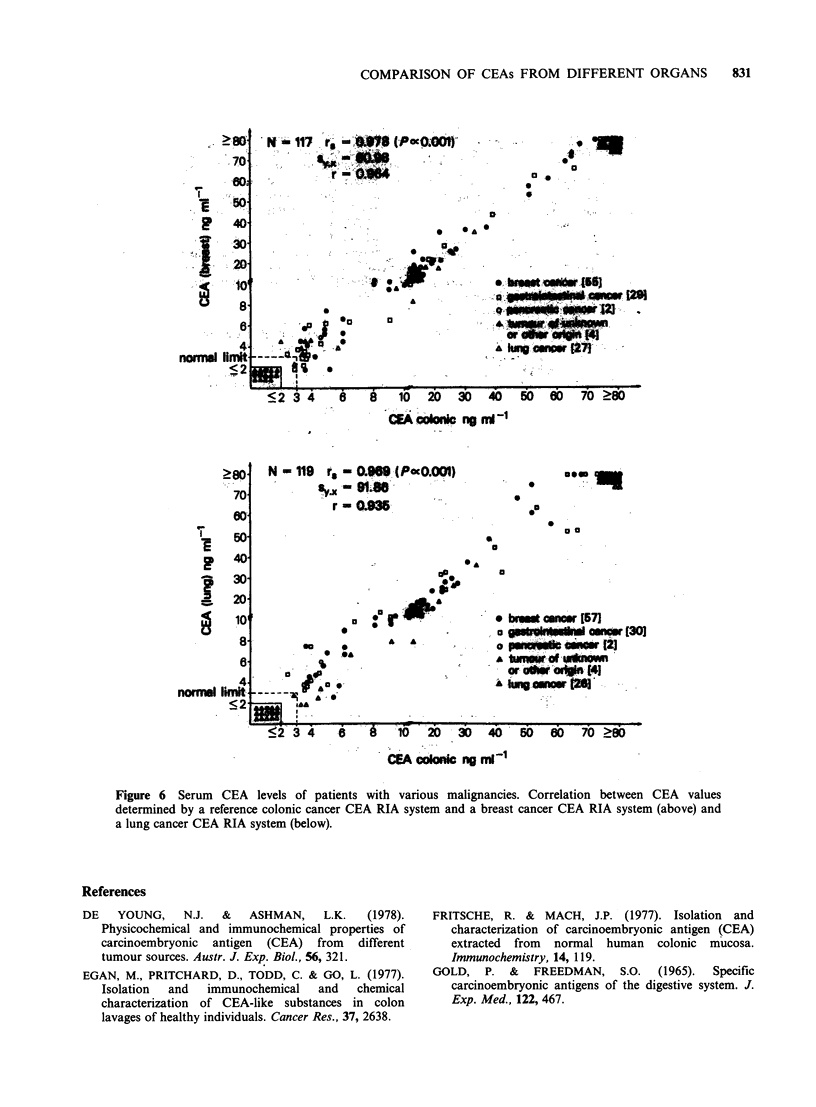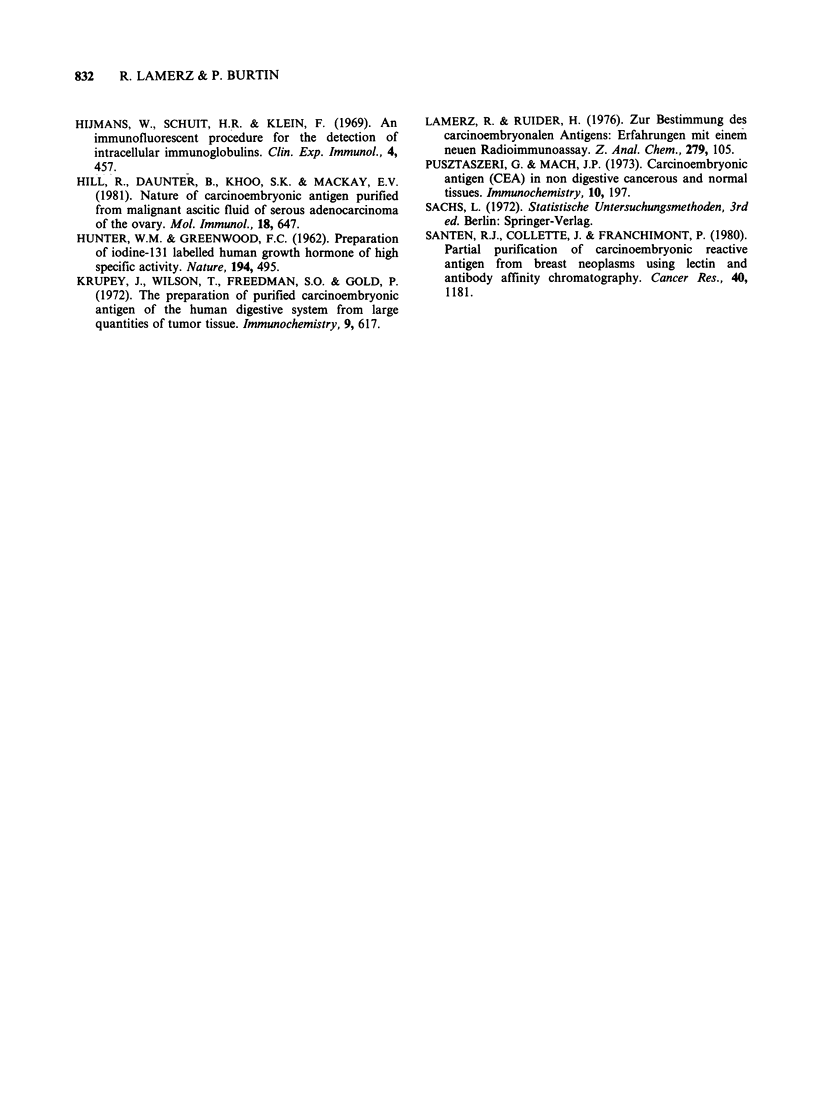# Immunological comparison of carcinoembryonic antigen (CEA) extracted from tumours of various organs: their use in radioimmunological CEA determinations.

**DOI:** 10.1038/bjc.1983.137

**Published:** 1983-06

**Authors:** R. Lamerz, P. Burtin

## Abstract

**Images:**


*Br. J. Cancer* (1983), **47**, 823–832

### Immunological comparison of carcinoembryonic antigen (CEA) extracted from tumours of various organs: their use in radioimmunological CEA determinations

R. Lamerz[1] & P. Burtin[2]

[1]*Med. Klinik II, Klinikum Grosshadern, University of Munich, Fed. Rep. Germany and* [2]*Laboratoire d'Immunochimie, I.R.S.C., Villejuif, France.*

**Summary** CEA was extracted by the perchloric acid method from primary adenocarcinomas of the colon and the ovary, from ascitic and pleural fluids from patients with pancreatic, lung and breast cancer, and from the cyst fluid of a benign ovarian cystadenoma. Further purification included gel filtration and affinity chromatography. Antisera against CEA from colon, breast, ovary, lung and pancreatic cancer were produced in rabbits. In double diffusion experiments, all these CEA samples showed a reaction of complete immunological identity with all the anti-CEA sera, whatever their origin. CEA from colon, breast, pancreas and ovary were labelled with $^{125}I$ and used in radioimmunological experiments. In a radioimmunological system where the tracer and the antiserum were constant, all the CEA used as standards gave parallel inhibition curves, having nearly identical slopes. This was another criterion of immunological identity. Sera of numerous cancer patients were assayed in several RIA systems, one of them being the classical system with colonic CEA as tracer and anti-colonic CEA as antiserum, the others being "organ specific" systems. The values obtained in these assays were found to be highly correlated: the rank coefficient of correlation between colonic and breast cancer RIA systems was $r_S = 0.96$, that between colonic and ovarian RIA systems, 0.92, that between colonic and pancreatic RIA systems, 0.97 and that between colonic and lung RIA systems 0.96. It is thus concluded that by use of different organ-derived CEA preparations and their corresponding polyclonal antisera, no significative differences in serum CEA levels may be expected. No evidence of organ specificity of serum CEA was found.

The carcinoembryonic antigen of the digestive system (CEA) was first described by Gold & Freedman (1965) as a tumour- and organ-specific antigen, as it was found only in the digestive adenocarcinomas. Later studies modified these conclusions, since CEA was found in the extracts of various non-digestive carcinomas (breast, lung, ovary, etc.) and of non-cancerous tissues (Pusztaszeri & Mach, 1973), especially of normal colonic mucosa. As for the latter, it was shown to be identical, biochemically and immunochemically, to the CEA extracted from colonic tumours (Fritsche & Mach, 1977; Egan *et al.*, 1977). A similar comparison between CEA extracted from non-digestive tumours and that of the colon would be very useful; better results might be expected when assaying CEA in sera of patients with non-digestive cancers by using CEA obtained from non-digestive organs and antisera prepared against these CEA. Such studies have not been conducted before, except by Santen *et al.* (1980) who prepared an antiserum to breast CEA. We report here the results of a study where we first prepared CEA from tumours of the ovary, breast, lung, pancreas and colon, followed by antisera against each of them. With these reagents, the problem of CEA organ specificity was investigated.

Correspondence: R. Lamerz.

Received 23 November 1982, accepted 22 March 1983.

### Materials and methods

### *Tumours/CEA source*

Surgical tumour specimens of primary colon cancer ($n = 110$) and primary ovarian cancer ($n = 10$) – both of the adenocarcinoma type – as well as ascitic and pleural fluids from patients with breast cancer ($n = 3$), lung cancer ($n = 1$), pancreatic cancer ($n = 1$), and a cyst fluid of a patient with benign ovarian cystadenoma were used for CEA extraction.

### *Serum specimens*

Serum samples of patients with breast cancer (207), ovarian cancer (42), pancreatic cancer (10), lung cancer (27), gastrointestinal cancer (70; 26 gastric, 44 colorectal), and with metastatic disease of unknown primary origin (11) were investigated.

### *CEA preparation*

(a) *Extraction.* Pooled tumour and single body fluid specimens were extracted by perchloric acid after the method of Krupey *et al.* (1972). In brief, thawed cryopreserved tumour specimens were dissected with scissors, transferred to a multimix machine and homogenised in saline in a Virtis Homogenizer and Potter–Elvehjeun apparatus. The tumour homogenate and body fluid specimens were then extracted in an equal volume of 1.2 M perchloric acid (PCA) and centrifuged. Within 45 min after the initiation of PCA extraction, the supernatant was dialysed against tap then distilled water without prior neutralisation and thereafter concentrated, ultra-filtered and lyophilised.

(b) *Purification of CEA.* Crude PCA extract (150–250 mg) was chromatographed by gel-filtration on Sephadex 6B (2.5 × 80 cm column; 0.05 M Tris-HCl buffer + 0.1 N NaCl, pH 6.0) to yield 4 fractions. The CEA-positive fraction(s) II and/or III detected by immunodiffusion and RIA were further separated on Sephadex G200 column (2.5 × 80 cm) to yield 3 peaks. The CEA-positive fraction I was purified by immunoadsorption on CNBr-activated Sepharose 4B column (2.0 × 20 cm) with coupled rabbit anti-CEA IgG (washing buffer: 0.15 M NaCl + 0.02 M Na-phosphate = PBS, pH 7.3; eluting buffer: PBS + 2.0 M KSCN). Further purification of the CEA-containing fraction was achieved by immunoadsorption on CNBr-activated Sepharose 4B coupled with rabbit anti-human serum IgG, where the non-adsorbed fraction was used. The purity of CEA fractions was checked by double immunodiffusion with antisera (polyvalent and monospecific) against contaminating serum and tissue proteins (see below) including anti-NCA-serum, as well as by RIA with anti-NCA-serum.

### *Antisera*

Female rabbits (New Zealand White) were immunised with purified CEA preparations (or normal human serum proteins) following the modified method of Hijmans *et al.* (1969) by two s.c. injections, each of 100–200 $\mu$g CEA (one week apart; the first one with Freund's complete adjuvant and the second with incomplete adjuvant) and 3 weeks later by 6 i.v. booster injections (2 per week with increasing volumes of 0.05, 0.1, 0.1, 0.2, 0.2 up to 0.4 ml from a total of 500 $\mu$g in 2 ml of alum hydroxide-precipitated CEA). Animals were sacrificed 2–3 months after the commencement of immunisation and the antisera absorbed by normal human serum (lyophilised, 10–30 mg ml$^{-1}$ antiserum), ABO red blood cells and by normal lung PCA extract (10–20 mg ml$^{-1}$). Antisera used for immunoadsorption and part of the antisera used for radioimmunoassay were finally purified by gradient elution on DEAE–Sephacel (buffer: 0.01 M Na-phosphate pH 8.0/0.5 M Na-phosphate, pH 4.5); only the IgG fractions were used.

For analytical purposes, two reference anti-colonic CEA antisera (one given by Dr. Hirata, Abbott Lab., the other from LKB), an anti-NCA-serum (No. 43), an antiserum against normal colon PCA extract and several commercial antisera against serum and tissue proteins (polyvalent: anti-human serum proteins; monospecific: anti-albumin, anti-acid alpha$_1$-globulin, anti-haptoglobin, anti-transferrin, anti-alpha$_1$-chymotrypsin, anti-ferritin, anti-lactoferrin, each of Behringwerke AG) were used.

### *CEA-analysis*

Crude extracts and CEA preparations as well as of the anti-CEA-antisera were analyzed by Ouchterlony double immunodiffusion on microslides and by immunoelectrophoresis.

### *CEA-radioimmunoassay*

CEA preparations used as tracer were labelled by the Chloramine T method (Hunter & Greenwood, 1962). Briefly, 5–15 $\mu$g of CEA were labelled with 300 $\mu$Ci Na$^{125}$I (Hoechst AG) for 30–45 min at room temperature. The tracer was separated from free iodine on Sephadex G50 fine (1 × 15 cm column) and further purified on Sephadex G200 (1.5 × 35 cm column). After establishing the relationship between immunoreactive antigen and antibody in dilution curves, the tracer was diluted to a working dilution and stored frozen in 10 ml portions until use.

The RIA was developed as double antibody test: the first antibody was anti-CEA IgG from the rabbit and the second an anti-rabbit-IgG-antiserum from a goat. For direct serum determinations, aliquots of CEA-free serum were added to standard dilutions (2.5–160 ng ml$^{-1}$).

### *Statistical analysis*

Statistical comparison of the regression coefficients of the different standard inhibition curves and the Spearman's rank coefficient of correlation of serum CEA determinations by different CEA radioimmunoassay systems were computed by conventional methods (Sachs, 1972).

### Results

### *CEA preparations and antisera*

CEA preparations and antisera are summarised in Table I. As shown in Figure 1, the different CEA

**Table 1** Antigens and antisera used in Ouchterlony and RIA experiments

| *Antigens* | *Antisera* |
|---|---|
| Colonic cancer CEA<br>CEA-CO | Anti-colonic cancer CEA<br>A-CEA-CO<br>Ouchterlony: LKB<br>RIA: Xaver IGG |
| Breast cancer CEA<br>CEA-MT | Anti-breast cancer CEA<br>A-CEA-MT No. 18 |
| Ovarian cancer CEA<br>CEA-OV | Anti-ovarian cancer CEA<br>A-CEA-OV No. 495 |
| Pancreatic cancer CEA<br>CEA-PA | Anti-pancreatic cancer CEA<br>A-CEA-PA No. 30 |
| Lung cancer CEA<br>CEA-BC | Anti-lung cancer CEA<br>A-CEA-BC No. 32 |

preparations – colonic, pancreatic, breast, ovarian and lung cancer CEA – give an immunological reaction of complete identity in double diffusion experiments – even by the use of different anti-CEA-antisera (A-CEA-CO, A-CEA-MT, A-CEA-OV, A-CEA-PA, A-CEA-BC). There is no spur of one over another CEA precipitin line using different antisera. In contrast, the slight doubling of some of the precipitin lines may be explained by an imbalance of the immunological reaction caused by the different concentrations of antigens and antibodies. When the different CEA preparations were tested against a monospecific anti-NCA serum, no precipitin lines were observed. In addition, absorption of the different anti-CEA-antisera specimens by CEA aliquots of different origins (0.1–0.4 mg ml$^{-1}$ antiserum) resulted in a complete disappearance of the precipitin lines.

*CEA labelling*

As the reference CEA we used our colonic CEA preparation of the CEA radioimmunoassay developed since 1975 (Lamerz & Ruider, 1976). During that period, more than 40 regular labelling experiments of colonic CEA have been carried out. Generally, the protein peak of the first column (Sephadex G50 fine) contained between 17.4 and 78.2% of radioactivity. Thirty-five–65% of the peak obtained by the second column (Sephadex G200) were used as tracer and yielded a specific activity between 6.7 and 12.5 $\mu$Ci $\mu$g$^{-1}$. It is noteworthy that only the high mol. wt. peak fraction of the second column turned out to be immunoreactive, the residual part containing inactivated material because of the chloramine T and/or iodination procedures. In this respect, results were comparable for CEA prepared from cancers of the breast, ovary, pancreas and lung, which had a specific activity of 9.4, 6.5, 6.9 and 15.4 $\mu$Ci $\mu$g$^{-1}$ respectively.

**Figure 1** Comparative reactivity in agar gels of CEA preparations of various origins and anti-CEA sera. (**a**) Comparison of CEA preparations from tumours of 5 different locations (CO, MT, BC, PA and OV) with antisera against CEA-CO and CEA-OV. Complete identity was obtained. (**b**) Comparison of CEA preparations from tumours of 5 different locations (CO, MT, BC, PA and OV) with antisera against CEA-PA and CEA-MT. (**c**) Comparative reactivity of CEA preparations from tumours of 4 different locations (CO, PA, OV and MT) with 5 different anti-CEA sera (A-CO, A-MT, A-OV, A-PA and A-BC).

*Antibody dilution curves*

All the anti-CEA sera were used to bind labelled CEAs of different origins. The binding capacity and the slope of the dilution curves were nearly the same when a given tracer was studied with different antisera: this is shown in Figure 2 where the binding of CEA-PA by antisera against CEA-CO, CEA-OV, CEA-MT and CEA-PA in comparison to an anti-NCA-serum is depicted. Similar results were obtained with other tracers. The antibody titre varied from one antiserum to another (50% end point of antibody dilution between 1/120,000 for A-CEA-CO, 1/15,000 for A-CEA-PA, A-CEA-MT, A-CEA-BC and 1/7,000 for A-CEA-OV).

*Standard inhibition curves*

The results of many experiments can be summarized as follows:

(1) Within one RIA system where the tracer and the first antibody were the same, different inhibitors, i.e. CEA identical to the tracer or originating from other organs, gave parallel inhibition curves: the slopes of these curves were the same. Figures 3 and 4 show some of the inhibition curves obtained when 2 standards (CEA from pancreas and colon) were compared in one RIA system. Other experiments gave similar results.

(2) When the same tracer was tested with 2 different antisera, one of them specific for the CEA originating from the same organ as the tracer, and the other prepared with a CEA of different origin, inhibition curves were obtained in each case with the 2 CEA standards corresponding to the antisera. The slopes of these curves, i.e. the B values of the logit-log regression lines reported in Table II were roughly identical (Expts. 1–4, 13–16, 29–32; N.S. - $P<0.05$), moderately different (Expts. 5–8, 9–12, 17–20; $P<10^{-2}$), or significantly different (Expts. 21–24, 25–28; $P<10^{-3}$). It is worth mentioning that the slope of the inhibition curves varied from 2.10 to 2.51, i.e. in a significant manner, when different preparations of CEA-CO were used as tracer.

(3) When different tracers were compared with the same antiserum, the slopes of the inhibition curves given by the same CEA sample were different. For instance, the anti-CEA-CO IgG was reacted with CEA samples prepared from various organs. The B values varied from 2.51 to 3.19 when CEA-PA and CEA-MT were respectively used as tracers, and CEA-CO as inhibitor. The differences were especially marked when anti-CEA-OV was used with CEA-CO and CEA-OV as tracers and standards: the B values were 2.02–2.01 with CEA-CO as tracer, and 2.83–2.92 with CEA-OV

**Figure 2** Antibody dilution curves of 4 different anti-CEA-antisera (A-CEA-CO, A-CEA-OV, A-CEA-MT and A-CEA-PA) and an anti-NCA-antiserum by use of a pancreatic cancer CEA tracer (CEA-PA* 10/80). Ordinate: bound activity (B) as per cent of total activity (T) after subtraction of non-specific binding (N) (=2%).

**Figure 3** CEA standard inhibition lines (logit-log plot) by use of one tracer (colonic cancer CEA), two antisera (above: anti-colonic CEA antiserum; below: anti-pancreatic CEA antiserum) and two standards (standard CEA-CO and CEA-PA). Ordinate: bound activity (B) as per cent of maximal binding ($B_0$) without inhibition and after subtraction of non-specific binding (N).

as tracer (Table II, expts. 3–4 and 7–8); and when anti-CEA-BC was reacted with CEA-CO and CEA-BC (B values 1.70–1.71 and 2.68–2.71 respectively as shown in Table II, expts. 27–28 and 31–32). In every case, CEA-CO gave lower B values than all the other CEAs.

All these data lead to the conclusion that the CEAs of different origins have the same inhibiting capacity, i.e. are antigenically identical. However, the labelling procedure might alter their reactivity in a manner which differs from one sample to another. It does not appear that the organ origin of CEA samples makes them more or less sensitive to this alteration, as colonic CEA itself gave variable results following iodination. Hence heterogeneity of the tracers plays a major role in the difference between inhibition curves. Finally, there is certainly some heterogeneity among the antisera which could also influence the variations between curves.

*Serum CEA determinations*

Sera of patients with gastrointestinal, breast, pancreas, ovary and lung cancer, or cancer of unknown primary origin were assayed for CEA content in different RIA systems: they were used to inhibit the binding of radiolabelled CEAs (of various origins) to their respective antisera. Figures 5 and 6 show that whatever the antiserum and the tracer, the CEA values were highly correlated. The rank coefficients of correlation were $r_S = 0.992$ ($P < 10^{-3}$; $n = 91$) when the ovarian and colonic RIA systems were compared, and $r_S = 0.979$ ($P < 10^{-3}$; $n = 126$) when the pancreatic and the colonic RIA systems were studied in parallel. The $r_S$ values were 0.978 ($P < 10^{-3}$; $n = 117$) for the comparison between breast and colonic RIA systems, and 0.969 ($P < 10^{-3}$; $n = 119$) for the comparison between the lung and colonic RIA systems (Figures 5 and 6).

**Table II** Parameters of standard inhibition curves dependent on different tracers, antisera and standards

| Tracer | Antiserum-concentration | Stand. +NHS inhib. | 50% point ng ml$^{-1}$ ($\bar{x}\pm s$) | $Y=A+B\cdot X$ A ($\bar{x}\pm s$) | B ($\bar{x}\pm s$) | Expt. No. |
|---|---|---|---|---|---|---|
| CEA-CO 4/75-7/80 ($n=33$) | A-CEA-CO (Xav) 1/80,000-1/120,000 | CEA-CO | 36.8±10 $n=230$ | 3.57±0.37 $n=238$ | −2.27±0.23 $n=238$ | |
| CEA-CO 3/80 | A-CEA-CO (Xav) 1/140,000 | CEA-CO | 26.7 | 2.99 | −2.10 | 1 |
| | | CEA-OV | 27.1 | 2.98 | −2.08 | 2 |
| | A-CEA-OV (495) 1/10,000 | CEA-CO | 20.6 | 2.65 | −2.02 | 3 |
| | | CEA-OV | 18.1 | 2.52 | −2.01 | 4 |
| CEA-OV 3/80 | A-CEA-CO (Xav) 1/50,000 | CEA-CO | 25.3 | 4.23 | −3.01 | 5 |
| | | CEA-OV | 25.8 | 4.15 | −2.96 | 6 |
| | A-CEA-OV (495) 1/3,000 | CEA-CO | 20.7 | 3.72 | −2.83 | 7 |
| | | CEA-OV | 19.3 | 3.76 | −2.92 | 8 |
| CEA-CO 10/80 | A-CEA-CO (Xav) 1/100,000 | CEA-CO | 35.3 | 3.89 | −2.51 | 9 |
| | | CEA-PA | 28.3 | 3.54 | −2.44 | 10 |
| | A-CEA-PA (30) 1/25,000 | CEA-CO | 44.6 | 3.66 | −2.22 | 11 |
| | | CEA-PA | 45.0 | 3.81 | −2.30 | 12 |
| CEA-PA | A-CEA-CO (Xav) 1/100,000 | CEA-CO | 28.9 | 3.67 | −2.51 | 13 |
| | | CEA-PA | 29.4 | 3.60 | −2.45 | 14 |
| | A-CEA-PA (30) 1/10,000 | CEA-CO | 41.7 | 3.75 | −2.31 | 15 |
| | | CEA-PA | 41.0 | 3.97 | −2.46 | 16 |
| CEA-CO 2/82 | A-CEA-CO (Xav) 1/120,000 | CEA-CO | 49.7 | 3.96 | −2.33 | 17 |
| | | CEA-MT | 50.7 | 3.99 | −2.34 | 18 |
| | A-CEA-MT (18) 1/12,000 | CEA-CO | 67.4 | 3.81 | −2.05 | 19 |
| | | CEA-MT | 76.1 | 3.66 | −1.98 | 20 |
| CEA-MT 2/82 | A-CEA-CO (Xav) 1/50,000 | CEA-CO | 28.5 | 4.53 | −3.11 | 21 |
| | | CEA-MT | 29.9 | 4.66 | −3.19 | 22 |
| | A-CEA-MT (18) 1/8,000 | CEA-CO | 46.4 | 4.06 | −2.44 | 23 |
| | | CEA-MT | 50.0 | 4.21 | −2.48 | 24 |
| CEA-CO 2/82 | A-CEA-CO (Xav) 1/120,000 | CEA-CO | 49.7 | 3.96 | −2.33 | 25 |
| | | CEA-BC | 54.0 | 4.07 | −2.35 | 26 |
| | A-CEA-BC (32) 1/12,000 | CEA-CO | 60.2 | 3.02 | −1.70 | 27 |
| | | CEA-BC | 65.8 | 3.11 | −1.71 | 28 |
| CEA-BC 2/82 | A-CEA-CO (Xav) 1/30,000 | CEA-CO | 36.0 | 4.29 | −2.76 | 29 |
| | | CEA-BC | 39.9 | 4.43 | −2.77 | 30 |
| | A-CEA-BC (32) 1/12,000 | CEA-CO | 46.2 | 4.47 | −2.68 | 31 |
| | | CEA-BC | 45.9 | 4.50 | −2.71 | 32 |

It is thus clear that when the serum of a non-digestive cancer (breast, lung, ovary) was assayed in (i) the conventional RIA, performed with colonic CEA as tracer and standard and anti-colonic CEA serum and (ii) "organ specific RIA", made with the CEA extracted from the same organ as that afflicted by the patient's cancer and the corresponding antiserum the CEA values thus obtained were the same or nearly identical in almost all cases.

### Discussion

It is clear that our results do not support the hypothesis of an organ specificity of CEA. All the CEA samples gave reactions of identity in Ouchterlony plates when reacted in criss-cross experiments with antisera against these CEA samples. Furthermore, the inhibition curves given by these CEAs were parallel: this is strong evidence

**Figure 4** CEA standard inhibition lines (logit-log plot) by use of one tracer (pancreatic cancer CEA), two antisera (above: anti-colonic CEA antiserum; below: anti-pancreatic CEA antiserum) and two standards (standard CEA-CO and CEA-PA). Ordinate and abscissa as Figure 3.

for the absence of any antigenic difference between them. Our conclusion is in agreement with the data obtained by De Young & Ashman (1978). These authors purified CEA from hepatic metastases of tumours originating in colonic, stomach, lung, pancreas and obtained also semipurified preparations from other metastases, derived from tumours of the breast, pancreas and oesophagus. In all these preparations, CEA had a mol. wt in the range of 200–300 K daltons and a similar amino-acid composition. The purified preparations did not contain NCA. All of them were used in comparative radioimmunoassays and gave parallel inhibition curves, thus showing no immunological difference. However, De Young & Ashman (1978) used only an antiserum prepared against colonic CEA in their comparative studies. They did not produce antisera against CEA from non-colonic tumours; therefore, they did not prove definitively that CEAs from different organs are immunologically identical.

More recently Hill *et al.* (1981) described the purification of a CEA sample from ascitic fluid of a serous cystadenocarcinoma of the ovary. This ovarian CEA gave a reaction of identity with a colonic CEA in agar immunodiffusion, but here again, only an antiserum against colonic CEA was used. No antiserum against the isolated ovarian carcinoma CEA was prepared. Comparison between ovarian and colonic CEA was sought with one antiserum only, thus allowing no definitive conclusions.

Contrary data were reported by Santen *et al.* (1980) who claimed to demonstrate an antigenic difference between breast and colonic CEA, on the grounds that in radio rocket experiments made with anti-breast tumour extract serum, breast CEA serial dilutions "gave greater changes in rocket heights than did purified colon CEA". However, their conclusions can be criticised because they did not take into account the possible presence of NCA in

**Figure 5** Serum CEA levels of patients with various malignancies. Correlation between CEA values determined by a reference colonic cancer CEA RIA system and an ovarian cancer CEA RIA system (above) and a pancreatic cancer CEA RIA system (below).

their rather crude preparations, nor that of antibodies cross-reacting with NCA in their anti-breast CEA serum. Thus, they could have measured both NCA and CEA in their radio rocket immunoelectrophoresis experiments.

As a whole we conclude that all the evidence is against organospecificity of CEA. Thus the assay of CEA in sera of patients with non-digestive carcinoma can be performed with a colonic CEA as tracer and an anti-colonic CEA serum as antibody source, as well as with reagents prepared with non-digestive CEAs: the results are the same in our experiments.

This research project was supported by Sander Foundation, Neustadt/Danube. The technical assistance of Mrs. A. Brandt and Miss E. Segura is gratefully acknowledged.

**Figure 6** Serum CEA levels of patients with various malignancies. Correlation between CEA values determined by a reference colonic cancer CEA RIA system and a breast cancer CEA RIA system (above) and a lung cancer CEA RIA system (below).

### References

DE YOUNG, N.J. & ASHMAN, L.K. (1978). Physicochemical and immunochemical properties of carcinoembryonic antigen (CEA) from different tumour sources. *Austr. J. Exp. Biol.*, **56**, 321.

EGAN, M., PRITCHARD, D., TODD, C. & GO, L. (1977). Isolation and immunochemical and chemical characterization of CEA-like substances in colon lavages of healthy individuals. *Cancer Res.*, **37**, 2638.

FRITSCHE, R. & MACH, J.P. (1977). Isolation and characterization of carcinoembryonic antigen (CEA) extracted from normal human colonic mucosa. *Immunochemistry*, **14**, 119.

GOLD, P. & FREEDMAN, S.O. (1965). Specific carcinoembryonic antigens of the digestive system. *J. Exp. Med.*, **122**, 467.

HIJMANS, W., SCHUIT, H.R. & KLEIN, F. (1969). An immunofluorescent procedure for the detection of intracellular immunoglobulins. *Clin. Exp. Immunol.*, **4**, 457.

HILL, R., DAUNTER, B., KHOO, S.K. & MACKAY, E.V. (1981). Nature of carcinoembryonic antigen purified from malignant ascitic fluid of serous adenocarcinoma of the ovary. *Mol. Immunol.*, **18**, 647.

HUNTER, W.M. & GREENWOOD, F.C. (1962). Preparation of iodine-131 labelled human growth hormone of high specific activity. *Nature*, **194**, 495.

KRUPEY, J., WILSON, T., FREEDMAN, S.O. & GOLD, P. (1972). The preparation of purified carcinoembryonic antigen of the human digestive system from large quantities of tumor tissue. *Immunochemistry*, **9**, 617.

LAMERZ, R. & RUIDER, H. (1976). Zur Bestimmung des carcinoembryonalen Antigens: Erfahrungen mit einem neuen Radioimmunoassay. *Z. Anal. Chem.*, **279**, 105.

PUSZTASZERI, G. & MACH, J.P. (1973). Carcinoembryonic antigen (CEA) in non digestive cancerous and normal tissues. *Immunochemistry*, **10**, 197.

SACHS, L. (1972). *Statistische Untersuchungsmethoden, 3rd ed.* Berlin: Springer-Verlag.

SANTEN, R.J., COLLETTE, J. & FRANCHIMONT, P. (1980). Partial purification of carcinoembryonic reactive antigen from breast neoplasms using lectin and antibody affinity chromatography. *Cancer Res.*, **40**, 1181.